# Supplementary material for: Assessment of malaria infection among pregnant women and children below five years of age attending rural health facilities of Kenya: A cross-sectional survey in two counties of Kenya
Source: PLoS One. 2021 Sep 16;16(9):e0257276. doi: 10.1371/journal.pone.0257276 (PMC8445417; doi:10.1371/journal.pone.0257276)
Supplement: S1 File — (DOCX) [file pone.0257276.s002.docx]

# SI: Study Questionnaire

# Client Exit Interview Questionnaire: For Caretakers of Children Under 5 Years and Pregnant Women [English Version]

**Section 1: Identifiers**

**ID Number………………………………………………………………………………[__|__]-[__|__]**

**Date……………………………………………………………………………...[__|__] [__|__] [__|__]**

**County Name……………………………………………...[_________________________________]**

**Facility Code….………………………………………………………………………[__|__|__]**

**Interviewer ID**…………………………………………………………………………………..[__|__]

**Time start**:………………………………………………………………………………………..[__|__]

**Time stop**:…………………………………………………………………………………….….[__|__]

1. What was the purpose of your visit to the health facility today?

Brought child< 5 years of age to the clinic? [Y/N].[__] **If yes complete sections 2-6 and section 8 to 9**

Woman brought self to the clinic? [Y/N]…………….[__] **If yes complete sections 2, sections 7 to 9**

**Section 2: Demographic characteristics of mothers/caregivers**

2.1 In what year were you born (Write down year)…………………………..……………[__|__|__|__]

**If cannot remember,** how old are you?........................................................................................[__|__]

2.2 What is the highest level of school you completed?

**Read out options and enter only one answer [__]**

1=No education

2=Primary

3=Secondary

4=Higher

5=Other (specify)[____________________]

6=Unknown

2.4 What is your marital status?

**Read out options, only enter one answer**……………………………………….………………...[__]

1=Currently married

2=Separated

3=Divorced

4=Widowed

5=Never married

2.5 What is your relationship with this child? **If self or pregnant women go to section 3**

**Read out options, only enter one answer**…………………………………………………………[__]

1=Mother

2=Father

3=Sibling

4=Grandmother

5=Relative

6=Other (specify)……………...…………………………………………….[____________________]

**Section 3: Attendance at Health Facility**

3.1 How did you get to the health facility/**mobile clinic**today? (Mode of transport)

**Read out options, only enter one answer**………………………………………………………….**[__]**

1=Walk

2= Bicycle

3=Bus

4=Private motor vehicle

5=Boat

6=Other (specify)[______________________]

3.2 How much did it cost to travel to the health facility?

**(KES)[Free=0000; DK=9999]…………………………………………………………...[__|__|__|__]**

3.3 How long did it take you to travel to the health facility today?

**Read out options, only enter one answer…………………………………………………………[__]**

1= Less 15 minutes

2=15 – 30 minutes

3= Between 30 minutes & 1 hour

4=Between 1 and 2 hours

5=More than 2 hours

**Section 4: Child < 5 years if age Medical History**

4.1 Child’s date of birth [dd-mm-yy]…………………………………………....[__|__]-[__|__]-[__|__]

4.2 Child’s Age (years-months)…………………………………………………………[__|__]-[__|__]

4.3 Child’s sex (M/F)………………………………………………………………………………..[__]

4.4 Did the health worker refer your child to another facility? (Y/N)………………………………[__]

4.5 Did the health worker admit your child to this facility? (Y/N)………………………………….[__]

**If yes to either 4.4 or 4.5 stop interview**

4.6 Is this your first visit to this health facility/**mobile clinic**with this child for this episode of illness? (Y/N) …..[__]

If no, continue with 4.7; if yes, skip to 4.8

4.7 How many times have you visited this health facility with this child for this illness episode?

Record number of times (including this visit)………………………………………………………[__]

4.8 Which complaints/symptoms prompted you to bring your child to this facility/mobile clinic (for first or return visit)?

**Mark only Y for complaints mentioned. DO NOT PROMPT**

4.8.1 How many days has your child had these symptoms?

Write duration (number of days) for each corresponding symptom.

| Symptom | Mark Y if mentioned | Duration [Number of days] |
| --- | --- | --- |
| Fever | [__] | [__\|__] |
| Cough | [__] | [__\|__] |
| Difficult breathing | [__] | [__\|__] |
| Not eating well | [__] | [__\|__] |
| Vomiting | [__] | [__\|__] |
| Diarrhoea | [__] | [__\|__] |
| Malaria | [__] | [__\|__] |
| Ear problem | [__] | [__\|__] |
| Convulsions | [__] | [__\|__] |
| Stomach ache | [__] | [__\|__] |
| Skin problem | [__] | [__\|__] |
| Headache | [__] | [__\|__] |
| Body/joint aches | [__] | [__\|__] |
| Other [specify][_____________________] | [__] | [__\|__] |
| Other [specify][_____________________] | [__] | [__\|__] |
| Other [specify][_____________________] | [__] | [__\|__] |

**Section 5: Diagnosis**

5.1 Did you or your child have blood taken from his/hers finger or heel for testing? [Y/N]……….[__]

**Section 6: Treatment**

6.1. What medication/drugs were you or your child given here at the **health/mobile clinic** today?

**Antimalarial drugs**

1. [__] Coartem/ AL/ACTArtefan

2. [__] Oral quinine

3. [__] Injectable quinine

4. [__] Artesunate

5. [__] Other (specify) [________________________________]

**Antibiotics**

1. [__] Pill/syrup

2. [__] Injection

**Other drugs**

1. [__] Panadol

2. [__] Aspirin

3. [__]Ibubrofen

4. [__] Other (specify) [________________]

5. [__] Other (specify) [________________]

**Section 7: ANC visit/pregnant woman**

7.1 I would like to confirm that you came to the **health facility** clinic for ANC and/or are pregnant? [Y/N]………..[__]

Is this your first pregnancy? [Y/N]………………………………………………………………..[__]

7.2 During this or previous visits have a health worker given or prescribed any antimalarial tablets to protect you from malaria?

**Read out options, only enter one answer…………………………………………………………[__]**

1=Yes, during this visit

2=Yes, previous visit

3= Yes, both previous and this visit

4=No-**Go to section 8**

5=Don’t know-**Go to section 8**

7.3 How many times during this pregnancy have you swallowed the tablets given to you to prevent malaria? **Read out options, only enter one answer……………………………………………………[__]**

1=Once

2=Twice

3=More than two times

4=Don’t know/cannot remember

7.4 Did the health worker ask you to take the drugs/tablets inform of him or her? [Y/N]……….…[__]

7.5 Did you take them? [Y/N]………………………………………………………………...……..[__]

7.6 If No, why?

7.7 Did the health worker explain to you how to take the tablets to prevent malaria?

**Read out options, only enter one answer………………………………………………………..……[__]**

1=Yes, this visit

2=Yes, previous visit

3=No

4=Don’t know

7.8 Did the health worker explain to you the number of doses of antimalarial drugs you need during this pregnancy? [Y/N]………………………………………………………………………………………[__]

7.9 Did the health worker explain why you need to use insecticide treated nets during pregnancy? [Y/N]………………………………………………………………………………………...…………[__]

**Section 8: Client satisfaction**

**I am going to ask questions about the services you received today at the health facility. I would like your honest opinion about the things we will talk about. This information will help improve prevention at treatment services at this facility.**

8.1 How long did you wait between the time you arrived at the facility and time you were able to see the health worker for consultation? **Saw health worker immediately=000 and Don’t know =9999**

Hours [__|__|__]

Minutes [__|__|__]

**8.2 Now I am going to ask about some common problems clients have at health facilities. As I mention each one, please tell me whether any of these were problems for you today, and if so, whether they were major or minor problems for you.**

8.2.1 Time you waited

**Read out options, only enter one answer………………………………………………………….[__]**

1=Major

2=Minor

3=No problem

9=Don’t know

8.2.2 Ability to discuss problems

**Read out options, only enter one answer……………………………………………………….…[__]**

1=Major

2=Minor

3=No problem

9=Don’t know

8.2.3 Amount of explanation you received about the medications you were given or prescribed

**Read out options, only enter one answer……………………………………………………….…[__]**

1=Major

2=Minor

3=No problem

9=Don’t know

8.2.4 Quality of the examination and treatment

**Read out options, only enter one answer…………………………………………………[__]**

1=Major

2=Minor

3=No problem

9=Don’t know

8.2.5 Privacy from having others see the examination

**Read out options, only enter one answer…………………………………………………[__]**

1=Major

2=Minor

3=No problem

9=Don’t know

8.2.6 Privacy from having others hear your consultation discussion

**Read out options, only enter one answer…………………………………………………[__]**

1=Major

2=Minor

3=No problem

9=Don’t know

8.2.7 Availability of malaria medicines at this facility

**Read out options, only enter one answer…………………………………………………[__]**

1=Major

2=Minor

3=No problem

9=Don’t know

8.2.8 The hours of service at this facility

**Read out options, only enter one answer…………………………………………………[__]**

1=Major

2=Minor

3=No problem

9=Don’t know

8.2.9 The number of days services are available to you

**Read out options, only enter one answer…………………………………………………[__]**

1=Major

2=Minor

3=No problem

9=Don’t know

8.2.10 The cleanliness of the facility

**Read out options, only enter one answer…………………………………………………[__]**

1=Major

2=Minor

3=No problem

9=Don’t know

8.2.11 How the staff treated you

**Read out options, only enter one answer…………………………………………………[__]**

1=Major

2=Minor

3=No problem

9=Don’t know

8.2.12 Cost for services or treatment

**Read out options, only enter one answer…………………………………………………[__]**

1=Major

2=Minor

3=No problem

9=Don’t know

8.2.13 Any problem you had today that I did not mention? (specify)

[_________________________________________________]

[_________________________________________________]

**Section 9: Additional services**

I would like to know from you, other than the services you received today or are provided at this health facility, are there any other services you would like included or provided for other illnesses in your community

1. Malaria diagnosis and treatment.....……………...………………..….[__]

IPT for pregnant women……………………………………….[__]

Provision of ITNS………………………………………….…..[__]

2. Diagnosis and treatment of worms………………….………………..[__]

3. Diagnosis and treatment of HIV……………….……………………..[__]

4. Provision of health education………………………………………...[__]

5. Others (specify)……………..[_________________________________]

[_________________________________]

[_________________________________]

**Thank you for your response**

# Client Exit Interview Questionnaire: For Caretakers of Children Under 5 Years and Pregnant Women [Kiswahili Translated Version]

**Section 1: Identifiers**

**ID Number……………………………………………………………………………...… [__|__]-[__|__]**

**Date………………………………………………………………………………... [__|__] [__|__] [__|__]**

**County Name……………………………………………....... [_________________________________]**

**Facility Code….………………………………………………………………………..……… [__|__|__]**

**Interviewer ID**…………………………………………………………………………………….. [__|__]

**Time start**: ………………………………………………………………………………………... [__|__]

**Time stop**: …………………………………………………………………………………….…... [__|__]

1. Ni nini madhumuni ya ziara yako kwahospitali/kliniki ya *Beyond Zero* leo?

Kuleta mtoto wa chini ya miaka tano katika hospitali kliniki? [Y/N]………... [__] **Kama ndio, kamilisha sehemu ya 2-6 na sehemu ya 8 hadi 9**

Mwanamke mwenyewe amejileta katika hospitali/kliniki? [Y/N]……………. [__]**Kama ndio, kamilisha sehemu ya 2, sehemu ya 7 hadi 9**

**Section 2: Demographic characteristics of mothers/caregivers**

2.1 Ulizaliwa mwaka gani (Write down year)…………………………..………………...… [__|__|__|__]

**Kama hawezi kumbuka,** una miaka mingapi?................................................................................ [__|__]

2.2 Ni ngazi gani ya juu ya shule uliokamilisha?

**Soma michaguo na uweke jibu moja tu [__]**

1=Hakuna elimu

2=Shule ya msingi

3=Sekondari

4=Elimu ya juu

5=Ingine (fafanua) [____________________]

6=Haijulikani

2.4 Hali yako ya ndoa?

**Soma michaguo na uweke jibu moja tu** ……………………………………….……………..…...... [__]

1=Ameolewa

2=Tengana/Wamewachana kwa muda

3=Talaka/taliki

4=Mjane

5=Hajawahi olewa

2.5 Uhusiano wako na huyu mtoto? **Kama ni yeye mwenyewe ama wanawake wajawazito enda kwa sehemu ya 3**

**Soma michaguo na uweke jibu moja tu** ……………………………………………….…………… [__]

1=Mama

2=Baba

3=Ndugu

4=Nyanya

5=Jamaa

6=Ingine (fafanua).……………………………………………………………. [____________________]

**Section 3: Attendance at Health Facility**

3.1 Ulitumia jinsi gani kufika katika kituo cha afya leo? (Utaratibu wa usafiri)

**Soma michaguo na uweke jibu moja tu** ………………………………………….…………...... **[__]**

1=Kutembea

2=Baiskeli

3=Basi

4=Gari ya kibinafsi

5=Mashua

6=Ingine (fafanua) [______________________]

3.2 Ilikugharimu kiasi gani kusafiri kuja kwa kituo cha afya?

**(KES)[Bure=0000; Sijui=9999]…………………………………………………….…....... [__|__|__|__]**

3.3 Ilikuchukuwa muda gani kusafiri kuja katika kituo cha afya leo?

**Soma michaguo na uweke jibu moja tu ……………………………………………….……..…..… [__]**

1=Chini ya dakika 15

2=Dakika 15-30

3=Kati ya dakika 30 na saa moja

4=Kati ya saa moja na mbili

5=Zaidi ya masaa mawili

**Section 4: Child < 5 years if age Medical History**

4.1 Siku ya kuzaliwa ya mtoto [dd-mm-yy]……………………………...……..... [__|__]-[__|__]-[__|__]

4.2 Umri wa mtoto (miaka-miezi)..…………………………………………...…...……… [__|__]-[__|__]

4.3 Jinsia ya mtoto (M/F)…………………………………………………………………...……..….. [__]

4.4 Je, mfanyakazi wa afya alirefari mtoto wako kwa hospitali nyingine? (Y/N)…….……………… [__]

4.5 Je, mfanyakazi wa afya alimlaza mtoto wako katika kituo hiki? (Y/N)……………………...…… [__]

**Kama ndio kwa 4.4 au 4.5 komesha mahojiano**

4.6 Je, hii ni ziara yako ya kwanza kwa kituo hiki cha afya na mtoto huyu kwa tukio hii ya ugonjwa? (Y/N) ………………………………………………………………………………………………….. [__]

**Kama hapana, endelea na 4.7; Kama ndio, enda kwa 4.8**

4.7 Ni mara ngapi wewe umetembelea hiki kituo cha afya na huyu mtoto kwa ajili ya tukio hili la ugonjwa?

Rekodi idadi ya nyakati (ikiwa pamoja na ziara hii)………………………………………....…… [__]

4.8 Ni malalamiko/dalili yapi ambayo yalikusababisha wewe kumleta mtoto wako kwa kituo hiki (ziara ya kwanza au ziara ya kurudia)?

**Weka ishara ya Y kwa malalamiko yaliotajwa. USIMKUMBUSHE.**

4.8.1 Mtoto wako amekuwa na dalili hizi kwa siku ngapi?

Andika muda (idadi ya siku) ziafikiane na kila dalili

| Dalili | Weka ishara ya Y kama imetajwa | Muda [Idadi ya siku] |
| --- | --- | --- |
| Joto mwilini | [__] | [__\|__] |
| Kukohoa/kikohozi | [__] | [__\|__] |
| Ugumu kupumua | [__] | [__\|__] |
| Hali/hakuli vizuri | [__] | [__\|__] |
| Kutapika | [__] | [__\|__] |
| Kuhara/ Kuharisha | [__] | [__\|__] |
| Malaria | [__] | [__\|__] |
| Shida ya sikio/masikio | [__] | [__\|__] |
| Kufifitika | [__] | [__\|__] |
| Kuumwa na tumbo | [__] | [__\|__] |
| Shida ya ngozi | [__] | [__\|__] |
| Kuumwa na kichwa | [__] | [__\|__] |
| Mwili/kiungo kuuma | [__] | [__\|__] |
| Ingine [fafanua][_____________________] | [__] | [__\|__] |
| Ingine [fafanua][_____________________] | [__] | [__\|__] |
| Ingine [fafanua][_____________________] | [__] | [__\|__] |

**Section 5: Diagnosis**

5.1 Je, wewe au mtoto wako mlichukuliwa damu kutoka kwa kidole chake au kisigino kwa ajili ya kupimwa? [Y/N]…….......................................................................................................................…. [__]

**Section 6: Treatment**

6.1. Ni matibabu/dawa gani ulipewa au mtoto alipewa katika kiliniki hii leo?

**Uliza mhojiwa akupe kadi au kitabu cha kliniki na uweke alama ya pata kwa madawa yalioandikwa na uliza kuona madawa alizopewa.**

**Madawa ya malaria**

1. [__] Coartem/ AL/ACTArtefan

2. [__] Oral quinine

3. [__] Injectable quinine

4. [__] Artesunate

5. [__] Other (specify) [________________________________]

**Kiuavijasumu**

1. [__] Pill/syrup

2. [__] Injection

**Madawa mengine**

1. [__] Panadol

2. [__] Aspirin

3. [__] Ibubrofen

4. [__] Other (specify) [________________]

5. [__] Other (specify) [________________]

**Section 7: ANC visit/pregnant woman**

7.1 Ningependa kuthibitisha kwamba umekuja kwahospitali ama kliniki kwa ajili ya ANC na/au ni mjamzito [Y/N]……………………………………………………………………………………………….….. [__]

Je, hii ni mimba yako ya kwanza? [Y/N]………………………...………………………………….... [__]

7.2 Wakati huu au ziara zilizopita mfanyakazi wa afya alikupa au kukuandikia tembe yoyote ya kukinga kutokana na malaria?

**Soma michango, weka jibu moja tu ……………………………………..…………...……..……… [__]**

1= Ndio, wakati wa ziara hii

2= Ndio, ziara iliopita

3= Ndio, zote**,** ziara iliopita na ziara hii

4= Hapana **Enda kwa sehemu ya 8**

5= Sijui **Enda kwa sehemu ya 8**

7.3 Ni mara ngapi umemeza tembe ulipewa kukinga dhidi ya malaria wakati wa ujauzito huu?

**Soma michango, weka jibu moja tu ……………………………………………....…………...…… [__]**

1=Mara moja

2=Mara mbili

3=Zaidi ya mara mbili

4=Sijui/hawezi kumbuka

7.4 Je, mfanyakazi wa afya alikuuliza umeze dawa/tembe mbele yake? [Y/N]…………………........ [__]

7.5 Je, ulimeza hayo tembe? [Y/N]…...…………………………...…………………………….....…. [__]

7.6 Kama hapana, kwa nini?

7.7 Je, mfanyakazi wa afya alikuelezea jinsi ya kutumia/kumeza tembe ya kukinga dhidi ya malaria?

**Soma michango, weka jibu moja tu ………………………………………………...……………… [__]**

1=Ndio, wakati wa ziara hii

2=Ndio, ziara iliopita

3=Hapana

4=Sijui

7.8 Je, mfanyakazi wa afya alikueleza idadi ya kipimo ya dawa ya malaria unahitaji wakati wa ujauzito huu? [Y/N]………………………………………………………………………………………..…… [__]

7.9 Je, mfanyakazi wa afya alikuelezea kwa nini unahitaji kutumia neti iliyotibiwa wakati wa ujauzito? [Y/N]…………………………………………………………………………….…………...…...…… [__]

**Section 8: Client satisfaction**

**Mimi ningependa kukuuliza maswali kuhusu huduma uliopokea hapa leo katikakituo cha afya ama kliniki ya *Beyond Zero*. Ningependa maoni yako juu ya mambo tutajadiliana. Habari hii itatusaidia kuboresha huduma ya kukinga na kutibu katika kituo hiki.**

8.1 Ulisubiri kwa muda gani kati ya wakati uliowasili katika kituo na wakati ulikuwa na uwezo wa kuona mfanyakazi wa afya kwa ajili ya kushauriana? **Aliona mfanyi kazi wa afya mara moja=000 na Sijui=9999**

Masaa [__|__|__]

Dakika [__|__|__]

**8.2 Sasa nitakuuliza kuhusu baadhi ya matatizo ya kawaida wateja hupata katika vituo vya afya. Nitataja kila moja, tafadhali niambie kama yeyote kati ya hizi zilikuwa matatizo yako leo, na kama hivyo, kama yalikuwa matatizo makubwa au madogo kwako?**

8.2.1Wakati ulisubiri

**Soma michango, weka jibu moja tu ……………………………………………...………….……... [__]**

1=Kubwa

2=Ndogo

3=Hakuna shida

9=Sijui

8.2.2 Uwezo wa kujadili matatizo

**Soma michango, weka jibu moja tu ………………………………………………...……………… [__]**

1=Kubwa

2=Ndogo

3=Hakuna shida

9=Sijui

8.2.3 Kiasi cha maelezo uliyopokea kuhusu matibabu uliyopewa au kuandikiwa

**Soma michango, weka jibu moja tu …………………………………………………...…………… [__]**

1=Kubwa

2=Ndogo

3=Hakuna shida

9=Sijui

8.2.4 Ubora wa uchunguzi na matibabu

**Soma michango, weka jibu moja tu ……………………………………………..……...………..… [__]**

1=Kubwa

2=Ndogo

3=Hakuna shida

9=Sijui

8.2.5 Usiri kutokana na wengine kuona uchunguzi

**Soma michango, weka jibu moja tu ………………………………………………….………..…… [__]**

1=Kubwa

2=Ndogo

3=Hakuna Shida

9=Sijui

8.2.6 Usiri kutokana na wengine kuskia mjadala wa kushauriana

**Soma michango, weka jibu moja tu …………………………………………………….………..… [__]**

1=Kubwa

2=Ndogo

3=Hakuna shida

9=Sijui

8.2.7 Upatikanaji wa madawa ya malaria katika kituo hiki

**Soma michango, weka jibu moja tu ………………………………………………………….…..… [__]**

1=Kubwa

2=Ndogo

3=Hakuna Shida

9=Sijui

8.2.8 Masaa ya huduma katika kituo hiki

**Soma michango, weka jibu moja tu ……………………………………………….……………..… [__]**

1=Kubwa

2=Ndogo

3=Hakuna shida

9=Sijui

8.2.9 Idadi ya masiku huduma inapatikana kwako

**Soma michango, weka jibu moja tu ………………………………………………….…………..… [__]**

1=Kubwa

2=Ndogo

3=Hakuna shida

9=Sijui

8.2.10 Usafi wa kituo

**Soma michango, weka jibu moja tu ………………………………………………….…………..… [__]**

1=Kubwa

2=Ndogo

3=Hakuna shida

9=Sijui

8.2.11Jinsi wafanyakazi walikuhudumia

**Soma michango, weka jibu moja tu ………………………………………………….…………..… [__]**

1=Kubwa

2=Ndogo

3=Hakuna shida

9=Sijui

8.2.12 Gharama ya huduma au matibabu

**Soma michango, weka jibu moja tu ………………………………………….…………………..… [__]**

1=Kubwa

2=Ndogo

3=Hakuna shida

9=Sijui

8.2.13 Tatizo lolote ulipata leo ambalo sijataja? (Fafanua)

[_________________________________________________]

[_________________________________________________]

**Section 9: Additional services**

Ningependa kujua kutoka kwako, mbali na huduma uliopokea leo au ambazo hutolewa katika kliniki ya *Beyond Zero,* kuna huduma zingine ambazo ungependa ziongezwe au zipeanwe kwa ajili ya magonjwa mengine katika jamii yako**?**

1. Utambuzi na matibabu ya Malaria.....……………...………………..……………………….. [__]

Kinga na matibabu ya malaria kwa wanawake wajawazito……….……………………. [__]

Ugawaji wa neti zilizo tibiwa…………………………………….……………………... [__]

1. Utambuzi na matibabu ya minyoo………………….……………………………………….... [__]
2. Utambuzi na matibabu ya Ukimwi..………….……………………………………………..... [__]
3. Utowaji wa elimu ya afya…………………………………………………………………...... [__]

5. Nyingine (fafanua)…………….. [_________________________________]

[_________________________________]

[_________________________________]

[_________________________________]

**Asante kwa majibu yako**

# Client Exit Interview Questionnaire: For Caretakers of Children Under 5 Years and Pregnant Women [Dholuo Translated Version]

**Section 1: Identifiers**

**ID Number…………………………………………………………………..…………… [__|__]-[__|__]**

**Date…………………………………………………………………………..…... [__|__] [__|__] [__|__]**

**County Name…………………………………………...…... [_________________________________]**

**Facility Code………………....…………………………...…………………………………… [__|__|__]**

**Interviewer ID**……………………………………………...…………………………………….. [__|__]

**Time start**:………………………………………………….…………………………………….. [__|__]

**Time stop**:……………………………………………………...……………………………….…. [__|__]

1. Ang’o momiyo ibiro e kar thieth kae kawuono?

Akelo nyathi mapok ochopo higni abich e klinik? [Y/N]……. [__] **Ka ee, tiek migawo mar 2 nyaka 6 gi migawo mar 8 nyaka 9**

Miyo owuon ema obiro e klinik? [Y/N]………………… …. [__]**Ka ee, tiek migawo mar 2, migawo mar 7 nyaka 9**

**Section 2: Demographic characteristics of mothers/caregivers**

2.1 No nyuoli iga mane (Write down year) ………………………………..……………..… [__|__|__|__]

**Ka ok onyal paro,** In gi igni adi? ......................................................................................................[__|__]

2.2 En okang mane ma malo maa i chope e somo/skul?

**Som kuom mondik, ndik dwuoko achiel …………………………………………..………………. [__]**

1= Ok asomo

2= Primari

3= Sekondari

4= Okang mamalo

5= Mamoko (ler) [____________________]

6= Ok Ong’ere

2.4 Be osega kendi/nyuomi?

**Som kuom mondik, ndik dwuoko achiel** ……………………………………...….………………... [__]

1= Okenda

2= Wawere matin

3= Wawere chuth

4= Chi liel

5= Pok o kenda

2.5 Wat mane moriwo u gi nyathini? **Ka en owuon kata miyo mayach dhie e migawo mar 3**

**Som kuom mondik, ndik dwuoko achiel** ………………………………………………...………… [__]

1= Min

2= Wuon

3= Owete

4= Dagi

5= Watgi

6= Mamoko (ler) ……………...…………………………………………….... [____________________]

**Section 3: Attendance at Health Facility**

3.1 Ibiro gang’o kar thieth kae kawuono? (Mode of transport)

**Som kuom mondik, ndik dwuoko achiel** …………………………………..………………………. **[__]**

1= Wuotho gi tielo

2= Ndiga

3= Bas

4= Nyamburko mar ng’ato

5= Yie

6= Mamoko (ler) [______________________]

3.2 Ni chulo pesa adi e wuoth mar biro kar thieth?

(KES) **[Nono=0000;Ok ong’eyo=9999]……………………...…….……………………... [__|__|__|__]**

3.3 Okawi seche adi chopo kar thieth kawuono?

**Som kuom mondik, ndik dwuoko achiel ………………………………………………………...… [__]**

1= Ok rom dakika 15

2= Dakika 15 – 30

3= Kind dakika 30 gi saa achiel

4=Kind saa 1 gi seche 2

5=Mokalo seche 2

**Section 4: Child < 5 years if age Medical History**

4.1 Iga mane onyuole nyathini[tarik-dwe-iga]…………………...……...... [__|__]-[__|__]-[__|__]

4.2 En gi igni adi (igni-dweche)……..…………………………………………………......... [__|__]-[__|__]

4.3 En wuoi/nyako (M/F)………………………………………………………………………......…. [__]

4.4 Be jathieth nonyisi ni mondo iter nyathini kar thieth machielo? (Y/N)………………………..… [__]

4.5 Be jathieth no ruako nyathi e wuod? (Y/N)……………………………………………………..... [__]

**Ka ee, e 4.4 kata 4.5 ichung nonro**

4.6 Be mae e biro ni mokuongo kar thieth kae gi nyathini e tuo ni? (Y/N) ……………………...…... [__]

**Ka oyoo, dhi mbele gi 4.7; ka ee, dhie e 4.8**

4.7 En didi misekelo nyathini kar thieth kae e tuo ni?

Record number of times (including this visit)……………………………………………………...… [__]

Dik dalo duto komedore gi biro ma sani…………………

4.8 En chandruok/ ranyisi mage mano mio ikelo nyathi kar thieth ka (ikele mokuongo kod duoke mar ariyo)?

**Ket manaY ne ranyisi mowach. KIK INYISE**

4.8.1 En odiochienge adi ma nyathini osebedo gi ranyisi gi?

Ndik thuolo (kar kwan mar odiochenge) kuom ranyisi kaka ranyisi

| Ranyisi | Ket Y kowach | Thulolo [Kar kwan mar odiochienge] |
| --- | --- | --- |
| Del maliet | [__] | [__\|__] |
| Fuolo/Ahonda | [__] | [__\|__] |
| Gamo yueyo | [__] | [__\|__] |
| Ok ochiem maber | [__] | [__\|__] |
| Ng’ok | [__] | [__\|__] |
| Diewo | [__] | [__\|__] |
| Malaria | [__] | [__\|__] |
| Shida mar it/Tuo mar it | [__] | [__\|__] |
| Rieruok | [__] | [__\|__] |
| Ich kach | [__] | [__\|__] |
| Shida mar pien (del) | [__] | [__\|__] |
| Wich bar | [__] | [__\|__] |
| Del/Fuonde maremo | [__] | [__\|__] |
| Mamoko[ler][_____________________] | [__] | [__\|__] |
| Mamoko[ler][_____________________] | [__] | [__\|__] |
| Mamoko[ler][_____________________] | [__] | [__\|__] |

**Section 5: Diagnosis**

5.1 Bende ne okaw rembi kata remb nyathini koa e lith lwete kata tiende mondo opim? [Y/N]......… [__]

**Section 6: Treatment**

6.1. En yath mane, mane in kata nyathini nomi e kar thieth kae kawuono?

**Penj jachiewre omi i kad kata bug klinik to iket alama mar yie ne yedhe mondik to be ipenj neno yedhe momiye.**

**Yedhe malaria**

1. [__] Coartem/ AL/ACTArtefan

2. [__] Oral quinine

3. [__] Injectable quinine

4. [__] Artesunate

5. [__]Mamoko (ler) [________________________________]

**Yedhe kute malaria manie remo**

1. [__] Pill/syrup

2. [__] Injection

**Yedhe mamoko**

1. [__] Panadol

2. [__] Aspirin

3. [__]Ibubrofen

4. [__]Mamoko (ler) [________________]

5. [__]Mamoko (ler) [________________]

**Section 7: ANC visit/pregnant woman**

7.1 Daher mar ng’eyo kane ibiro e od thiethe klinik mar mine mayach/ koso ipek? [Y/N]………………...….... [__]

Mae ichni/ijni e mokuongo? [Y/N]…………………………………………………………………….. [__]

7.2 Kuom limbe ni masani kata e kinde mosekalo be jathieth nosemiyi yath moro amora mageng’o malaria?

**Som kuom mondik, ndik dwuoko achiel ………………………………………...………....……… [__]**

1= Ee, e limbe masani

2= Ee, limbe mosekalo

3= Ee limbe tee mosekalo kod limbe masani.

4= Dawe/Ooyo-**Dhi e migawo mar 8**

5= Ok ong’eyo- **Dhi e migawo mar 8**

7.3 Ekinde mane in gi ijni, isemuonyo yedhe momiyi mag geng’o malaria nya didi?

**Som kuom mondik, ndik dwuoko achiel …………………………………………..………….…… [__]**

1= Dichiel

2= Dirio

3= Mokalo nyadirio

4= Akia/ Ok anyal paro

7.4 Be jathieth no nyisi ni imuony yath ee mbele ne? [Y/N]………………………………………… [__]

7.5 Be ni mwuonyo gi? [Y/N]…………………………………………………………………..…….. [__]

7.6 Ka oyoo, ang’o momiyo?

7.7 Be jathieth no leroni kaka itiyo gi yath mondo ogeng’ malaria?

**Som kuom mondik, ndik dwuoko achiel ………………………………………………………...… [__]**

1= Ee, limbe masani

2= Ee, limbe mosekalo

3= Dawe/Ooyo

4= Ok ong’eyo

7.8 Be jathieth ne oleroni kar dose adi mag yedhe malaria monego bedni iti godo e kinde ma ipek? [Y/N]…………………………………………………………………………………………..……… [__]

7.9 Be jathieth oleroni gima omiyo onego iti gi net mothiedhi gi yath mar suna e kinde ma ipek? [Y/N]…………………………………………………………………………………...…...………… [__]

**Section 8: Client satisfaction**

**Adwaro penjo kit thieth mane iyudo kawuono e klink mamoko gi mar *Beyond Zero*. Daher mondo ilerna adiera e weche ma wabiro wacho. Weche gi biro konyo ebero yore mag geng’o kod thieth e kar thieth kae.**

8.1 Nirito kuom thuolo maromo nadi nyaka ne ichop e kar thieth gi sama ni nyalo neno jathieth mondo ulalru? **Aneno jathieth sano =0000 and Ok ong’eyo =9999**

Seche [__|__|__]

Dakika [__|__|__]

**8.2 Koro abiro penji kuom chandruok/shida makawaida ma ji ni godo e ut thieth. Sama awacho achiel ka achiel, yie mondo inyisa kaponi in bende iyudo chandruok/shida gi kawuono, to ka kamano, ne gin maduongo koso matin ni?**

8.2.1 Seche mirito

**Som kuom mondik, ndik dwuoko achiel …………………………………...…………………...…. [__]**

1= Maduong

2= Matin

3= Onge shida

9= Ok Ong’eyo

8.2.2 Nyalo loso e wi chandruok/shida

**Som kuom mondik, ndik dwuoko achiel …………………………………………...………...….… [__]**

1= Maduong

2= Matin

3= Onge shida

9= Ok Ong’eyo

8.2.3 Thoth mar ler maniyudo kuom thieth mane omiyi kata mondikni.

**Som kuom mondik, ndik dwuoko achiel …………………………………………………...…....… [__]**

1= Maduong

2= Matin

3= Onge shida

9= Ok Ong’eyo

8.2.4 Ber mar pim kod thieth

**Som kuom mondik, ndik dwuoko achiel ………………………………………………....…...…… [__]**

1= Maduong

2= Matin

3= Onge shida

9= Ok Ong’eyo

8.2. Bet kamoro kama jokma moko ok ne sama itimoni pim/siri

**Som kuom mondik, ndik dwuoko achiel ………………………………………………….......…… [__]**

1= Maduong

2= Matin

3= Onge shida

9= Ok Ong’eyo

8.2.6 Bet kamoro kama joma moko kik winj kaka ulalru gi jathieth/siri

**Som kuom mondik, ndik dwuoko achiel ………………………………………………………...… [__]**

1= Maduong

2= Matin

3= Onge shida

9= Ok Ong’eyo

8.2.7 Yudruok mag yedhe mag malaria e kar thieth kae

**Som kuom mondik, ndik dwuoko achiel ………………………………………………………...… [__]**

1= Maduong

2= Matin

3= Onge shida

9= Ok Ong’eyo

8.2.8 Seche mag tich e kar thieth kae

**Som kuom mondik, ndik dwuoko achiel ……………………………………………...………....… [__]**

1= Maduong

2= Matin

3= Onge shida

9= Ok Ong’eyo

8.2.9 Odiochenge adi ma kony yudre ni

**Som kuom mondik, ndik dwuoko achiel ………………………………………………………...… [__]**

1= Maduong

2= Matin

3= Onge shida

9= Ok Ong’eyo

8.2.10 Ler mar kar thieth

**Som kuom mondik, ndik dwuoko achiel ………………………………………………………...… [__]**

1= Maduong

2= Matin

3= Onge shida

9= Ok Ong’eyo

8.2.11 Kaka jotich norwako u

**Som kuom mondik, ndik dwuoko achiel …………………………………………………...…....… [__]**

1= Maduong

2= Matin

3= Onge shida

9= Ok Ong’eyo

8.2.12 Chudo mar kony kata thieth

**Som kuom mondik, ndik dwuoko achiel …………………………………………………...…....… [__]**

1= Maduong

2= Matin

3= Onge shida

9= Ok Ong’eyo

8.2.13 Chandruok/shida moro amora madi ne iyudo kawuono to ok awacho? (Ler) [_________________________________________________]

[_________________________________________________]

**Section 9: Additional services**

Daher mar ng’eyo koa kuomi ni kopogore kod thieth mane iyudo kawuono kata maichiwo e kar thieth, bende nitie thieth mamoko ma diher ni mondo omedie kata ochiw kuom tuoche mamoko e oganda u?

1. Pim kod thieth mar malaria.....……………...………………………………………..………….…. [__]

Geng’o malaria kuom mine mayach………………………………….…………………...…. [__]

Miyo ji net mosethiedh gi yath mar suna ……………………………………...…………….. [__]

2. Pim kod thieth mar kute………………….…………………………………………………...…..... [__]

3. Pim kod thieth mar Ayaki ……………….………………………………………...……………..... [__]

4. Chiwo puonj kuom weche mag ngima……………………………………………………….…...... [__]

5. Mammoko (ler) …………….. [_________________________________]

[_________________________________]

[_________________________________]

[_________________________________]

**Ero kamano kuom dwoko mari**
