# Supplementary material for: Assessment of malaria infection among pregnant women and children below five years of age attending rural health facilities of Kenya: A cross-sectional survey in two counties of Kenya
Source: PLoS One. 2021 Sep 16;16(9):e0257276. doi: 10.1371/journal.pone.0257276 (PMC8445417; doi:10.1371/journal.pone.0257276)
Supplement: Attachment — Submitted filename: Response to reviewers.docx [file pone.0257276.s004.docx]

Note: Throughout this “response to reviewer” document we give line reference to the manuscript with trackchanges

Journal Requirements:

When submitting your revision, we need you to address these additional requirements.

1. Please ensure that your manuscript meets PLOS ONE's style requirements, including those for file naming. The PLOS ONE style templates can be found at

<https://journals.plos.org/plosone/s/file?id=wjVg/PLOSOne_formatting_sample_main_body.pdf> and

<https://journals.plos.org/plosone/s/file?id=ba62/PLOSOne_formatting_sample_title_authors_affiliations.pdf>

 >> We have ensured that our manuscript meets PLOS ONES’s style.

2. Thank you for including your ethics statement:  "The study protocol received ethical approval from KEMRI’s Scientific and Ethics Review Unit (SERU No. 3252). Additional approval was provided by the county-level health authorities after they were appropriately briefed about the study. At health facility level, individual consent for pregnant women and written parental consent for children below five years was obtained.".

Please provide additional details regarding participant consent. In the ethics statement in the Methods and online submission information, please ensure that you have specified what type you obtained (for instance, written or verbal, and if verbal, how it was documented and witnessed). If your study included minors, state whether you obtained consent from parents or guardians. If the need for consent was waived by the ethics committee, please include this information.

Once you have amended this/these statement(s) in the Methods section of the manuscript, please add the same text to the “Ethics Statement” field of the submission form (via “Edit Submission”).

For additional information about PLOS ONE ethical requirements for human subjects research, please refer to <http://journals.plos.org/plosone/s/submission-guidelines#loc-human-subjects-research>.

 >> We have now moved the “ethics statement” to the methods section of the manuscript, see lines 241-245. We have also amended the online submission form with the same statement.

>> In the ethics statement, we have now stated clearly that written consent was obtained from pregnant women and written consent was obtained from the parents/guardians of the children below five years, please see lines 241-245.

3. We suggest you thoroughly copyedit your manuscript for language usage, spelling, and grammar. If you do not know anyone who can help you do this, you may wish to consider employing a professional scientific editing service.

>> We have copyedited this manuscript to correct for language use, spelling and grammar.

Whilst you may use any professional scientific editing service of your choice, PLOS has partnered with both American Journal Experts (AJE) and Editage to provide discounted services to PLOS authors. Both organizations have experience helping authors meet PLOS guidelines and can provide language editing, translation, manuscript formatting, and figure formatting to ensure your manuscript meets our submission guidelines. To take advantage of our partnership with AJE, visit the AJE website (http://learn.aje.com/plos/) for a 15% discount off AJE services. To take advantage of our partnership with Editage, visit the Editage website ([www.editage.com](http://www.editage.com)) and enter referral code PLOSEDIT for a 15% discount off Editage services.  If the PLOS editorial team finds any language issues in text that either AJE or Editage has edited, the service provider will re-edit the text for free.

Upon resubmission, please provide the following:

- The name of the colleague or the details of the professional service that edited your manuscript

>> The document was copy-edited by our colleague Mr. Benerd Chieng

- A copy of your manuscript showing your changes by either highlighting them or using track changes (uploaded as a *supporting information* file)

>> The track changes document was the one which was copy-edited before we commenced the revision and it is the same one that shows our response to reviewers

- A clean copy of the edited manuscript (uploaded as the new *manuscript* file)

>> We have attached one final clean copy being for both copy-editing and review response changes

4. Please include additional information regarding the survey or questionnaire used in the study and ensure that you have provided sufficient details that others could replicate the analyses. For instance, if you developed a questionnaire as part of this study and it is not under a copyright more restrictive than CC-BY, please include a copy, in both the original language and English, as Supporting Information.

 >> Details regarding the study questionnaire has been indicated in lines 180-185, and a copy of the questionnaire provided as a supplementary material.

5. Please confirm if your survey or questionnaire was pre-tested or validated. If these did not occur, please provide the rationale for not doing so.

>> We have mentioned that our questionnaire was pre-tested prior to the study, see line 181.

 6. Your ethics statement should only appear in the Methods section of your manuscript. If your ethics statement is written in any section besides the Methods, please move it to the Methods section and delete it from any other section. Please ensure that your ethics statement is included in your manuscript, as the ethics statement entered into the online submission form will not be published alongside your manuscript.

  >> We have now moved the “ethics statement” to the methods section of the manuscript, see lines 241-245. We have also amended the online submission form with the same statement.

Reviewers' comments:

Reviewer's Responses to Questions

**Comments to the Author**

1. Is the manuscript technically sound, and do the data support the conclusions?

The manuscript must describe a technically sound piece of scientific research with data that supports the conclusions. Experiments must have been conducted rigorously, with appropriate controls, replication, and sample sizes. The conclusions must be drawn appropriately based on the data presented.

Reviewer #1: Yes

Reviewer #2: Partly

2. Has the statistical analysis been performed appropriately and rigorously?

Reviewer #1: No

Reviewer #2: No

3. Have the authors made all data underlying the findings in their manuscript fully available?

The [PLOS Data policy](http://www.plosone.org/static/policies.action#sharing) requires authors to make all data underlying the findings described in their manuscript fully available without restriction, with rare exception (please refer to the Data Availability Statement in the manuscript PDF file). The data should be provided as part of the manuscript or its supporting information, or deposited to a public repository. For example, in addition to summary statistics, the data points behind means, medians and variance measures should be available. If there are restrictions on publicly sharing data—e.g. participant privacy or use of data from a third party—those must be specified.

Reviewer #1: No

Reviewer #2: Yes

4. Is the manuscript presented in an intelligible fashion and written in standard English?

PLOS ONE does not copyedit accepted manuscripts, so the language in submitted articles must be clear, correct, and unambiguous. Any typographical or grammatical errors should be corrected at revision, so please note any specific errors here.

Reviewer #1: Yes

Reviewer #2: Yes

5. Review Comments to the Author

Please use the space provided to explain your answers to the questions above. You may also include additional comments for the author, including concerns about dual publication, research ethics, or publication ethics. (Please upload your review as an attachment if it exceeds 20,000 characters)

Reviewer #1: The manuscript by Okoyo et al entitled, “Assessing malaria infection among pregnant women and children below five years of age attending rural health facilities of Kenya: A cross-sectional survey in two counties of Kenya” describes the prevalence of Plasmodium falciparum infection in pregnant women and children under five years of age using results from cross-sectional surveys conducted at rural health facilities in two Kenyan counties. The primary finding was a PCR positivity rate for malaria of 34.1% in pregnant women and 22.0% for children under five, with positivity varying by geographic location. A majority of pregnant women (86.4%) reported attending ANC at least once, though 62.1% did not have their first visit until their third trimester, which explains why most women (75%) did not receive their first IPTp dose until their second or third trimester.

>> This is a good summary of our results, thank you.

The authors thoroughly describe the methods employed for sample processing and examination and report extensively on the prevalence of Plasmodium falciparum infection in their study population. The analysis is limited in its assessment of risk factors and is primarily focused on descriptive statistics. This study contributes to the knowledge of malaria prevalence in these counties, providing a baseline against which to measure the effect of future malaria programs targeted towards pregnant women and children under five.

>> Thanks for this fair assessment of our study, we have endeavored to improve it further based on the review and editorial comments.

Major comments:

1. Further describe the knowledge gaps this manuscript aims to address. In the Introduction, there is substantial information about the status of malaria prevention efforts in Kenya, but limited explanation of what sets the study apart from other malaria surveys in the country. Additionally the abstract should state why the study was done.

>> Thanks for this comment. We have added a few sentences to explain further the gap and our contribution, please see lines 100-105.

>> We have indicated why the study was done in the abstract section, see lines 41-45.

2. The analysis of ANC utilization and SP-IPTp uptake should consider the trimester of the participants. For example in Line 246: Was the 8.2% of ANC attendees who completed ANC visits counted only from women late in the third trimester?

>> This was counted among all study-enrolled women, but women who were found to have completed their ANC visits were mostly in their third trimester.

If women are in their first or second trimester they would not be expected to have 4 ANC visits completed. Nor should women in the first trimester have received SP-IPTp.

>> It is true that women in first or second trimester would not be expected to have complete ANC visits, but the idea here was to find out at what stage of pregnancy do women start ANC visits and also start receiving IPTp-SP doses and compare this with the national guidelines.

It is also not clear if women were recruited from the ANC clinic how could any of the participants could not be enrolled in antenatal care? This only seems possible if the study visit took place prior to the clinic visit and the visit that day was not included. One way to clarify this might be to stratify the current Table 2 by trimester of the woman enrolled. How to take this into account in the univariate and multivariable analyses should also be considered (see #3).

>> Women were recruited immediately they arrived at the facility and prior to being attended to at the clinic, see lines 190-191. The ANC visit that day was not counted.

3. The multivariable analysis should be clarified and consider expanding. Specifically, were separate multivariable analyses conducted for children and pregnant women? If so, this should be clarified in the methods and the table. If not, then there should be separate analyses for these two group. Additional co-variates should be considered for the analysis of children. Was data collected on reprted net use? Are there trends in age? Were other socio-economic data collected? The description of which variabes were included in the multivariable analysis should also be clarified.

>> Thanks for this comment. Multivariable analysis (Table 4) was conducted among pregnant women only due limited variables among children. We have now put an explanation in lines 263-265 as well as lines 376-377. At this stage we cannot consider more covariates for children since very limited information was collected among them. We did not collect detailed information on net use or detailed socio-economic data. We have now clarified which variables were included in the multivariable analysis, see lines 377-378.

4. The manuscript is very well-written, but the text could be tightened up so that the detail of the findings are more easily accessible to the reader. The study design and results are very straightforward and do not require the length and level of detail in the text to convey the relevant messages. There substantial repetition of data in text and tables. For example, the paragraph in lines 210-8 could be removed as all of the data is presented in the table. Also, unless interventions are likely to be tailored at the sub-county level, the variability at the sub-county level could be acknowledged in the text, but the details and the analysis could be moved to a supplement.

>> Thanks for this comment and for the compliment. We believe that by revising this manuscript as per the editor’s and reviewers’ comments we have improved on its writing. We have tried to delete throughout the text instances where there are substantial repetitions. In the cited paragraph, we only summarized the key results with details left in the table.

Minor comments:
1. Abstract: Add that the study also assessed ANC utilization as stated in the last sentace of the main text Introduction.

>> Thanks for this suggestion, we have now added this information.

2. Introduction: Consider updating the World Malaria Report references to the more recent reports.

>> In this study, we have only cited two World Malaria Reports of 2015 and 2017. Given that our study collected data in 2017, we believe that those malaria reports are recent and more comparable to the data collection period.

3. Introduction: Check line 113, were only 8% of malaria cases in Kenya in under 5s? It seems likely that either the wording is unclear or the number is incorrect.

>> This must have been oversight, we have now corrected the figure to 80%.

4. Methods: How were the women and children chosen? Were they pairs, meaning the pregnant women were there with an older child and so they were sampled together? If this were the case, primigravidae would be underrepresented or absent. Also because of natural birth spacing, younger children would be underrepresented.

>> The study did not pair pregnant women with children below five years. These were separate groups of participants who were coming to the health facility. But it was possible that some pregnant women were accompanied with their children who were below five years to the clinic. However, their data were not paired. We have now clarified this in the text, please see lines 188-190.

5. Methods: For the sample size calculations, was there a target precision of estimates that was desired?

>> We have now detailed the sample size calculation procedure in lines 155-163. We considered a margin of error of 5%.

6. Methods: Please include the malaria transmission season of May and July in this area of Kenya.

>> We have now mentioned this in lines 172-174 and lines 464-467.

7. Methods: Were the surveys based on a validated questionnaire, e.g. the Malaria Indicator Survey? If so, consider referencing the validated tool.

>> Yes, we have now referenced the validated tool, please see lines 183-184

8. Methods: Were the microscopy slides read by two readers or just one?

>> We have now indicated that the microscopy slides were read independently by two technicians, please see lines 208-210.

9. Methods: Pf infection is defined as requiring both positive microscopy and PCR. However, much of the statistical analysis uses PCR positivity as the outcome. Consider clarifying the definitions of infection in the methods. Based on the data in presented in Table 1, there were many microscopy false positives or pcr false negatives (microscopy positivity > pcr positivity) and as expected some submicroscopic infection (pcr positivity > being both pcr and microscopy positive). Given that the results are primarily based on PCR, I assume the authors are more confident in their PCR data. If so, they should be more clear about their interpretation of their results and not leave the reader to make their own conclusions.

>> Thanks for this comment. We had stated that Pf infection was first defined by microscopy then confirmed by PCR (see lines 251-253). As much as many of the malaria results in the text were referring to PCR results we had also stated and compared it with microscopy both in the text and tables. We have now put more clarification here, please see lines 253-254. Additionally, we have now outlined the limitations of microscopy technique that made us compared its results with PCR and subsequently relied more on PCR results, see lines 469-478.

10. Methods: Please clarify the term “minimum generated variables” in line 194.

>> By minimum generated variables we mean that out of all variables subjected to univariable analysis, a section of them meeting a pre-specified p-value (in this case 0.3), were selected in a sequential variable selection method for multivariable analysis. These set of new variables considered for multivariable analysis are what we referred to as “minimum generated variables”.

11. Results: Consider including a standard Table 1 showing the demographic characteristics of the study population overall and by county. In this table and in the text, months should be the unit of age for children.

>> Our current table 1 acts as the demographic table as well and we have now referenced the first paragraph of the results section to table 1. Additionally, we have sub-grouped the children ages to <2 years, 2-3 years and 4 - <5 years, to give more information about children ages.

12. Results: Could the month difference in the survey dates explain some of the differences in prevalence between the counties? If so, this might be acknowledged in the discussion.

>> Thanks for this comment. We have now acknowledged it in our limitations, see lines 438-440.

13. Results and Tables: Please define all the variables used to calculate adjusted ORs

>> Thanks for this comment. We have now defined these variables in lines 464-467.

14. Table 2: Please add p-values to show statistically different distributions of variables by county. “Median gestational age in weeks (quartiles)” for each trimester is not useful and could be removed.

>> Thanks for this suggestion. We have now included the p-values (see table 2), improved the analysis methods section (see lines 268-270), and amended results reporting the p-values (see lines 316-340). Additionally, we have now deleted the median gestational age as suggested.

15. Results: The paragraph in lines 266-272 is methods, not results.

>> Thanks for this observation, we have now moved it to the section on data management and analysis (lines 256-267).

16. Discussion: Consider adding discussion of barriers to seeking care in these counties which need to be addressed based on this data.

>> Thanks for this suggestion, we have now added a discussion on the barriers, please see lines 442-457.

17. Discussion: Consider adding details about the limitations of the microscopy results if in fact the authors have concerns about their utility.

>> Thanks for this suggestion, we have now added statement about limitations of microscopy, please see lines 469-478.

18. Discussion/Conclusion: Consider speculating on what exactly should be done differently based on these results.

>> Thanks for this suggestion, we have now added a statement on the issue, please see lines 490-494.

Editorial comments: This manuscript is well-written, a few small comments.
1. Abstract: In the Methods it is not clear if cluster random sampling was used to select the participants or the health centers. Please make the language clearer.

>> We have now clarified in the methods that cluster sampling was used for selecting participants only, while health facilities we randomly sampled from the available facilities in each sub-county, see “study design and sampling” sub-section.

2. Abstract: Line 49, high”er” not highest

>> We have corrected this appropriately.

3. Abstract: Consider changing “transmission” in line 56 to “prevalence”

>> We have changed this appropriately.

Reviewer #2: This study describes malaria status in pregnant women and young children in two counties in Kenya. The manuscript needs major revisions with attention in particular to points like inclusion and exclusion criteria of study participants, and sample size calculation and why a cluster random design was chosen. These aspects are weakly given or lacking in the manuscript.

>> Thanks for this suggestion, we have now revised the methods section to address these issues, please see lines 141-165.

6. PLOS authors have the option to publish the peer review history of their article ([what does this mean?](https://journals.plos.org/plosone/s/editorial-and-peer-review-process#loc-peer-review-history)). If published, this will include your full peer review and any attached files.


If you choose “no”, your identity will remain anonymous but your review may still be made public.


**Do you want your identity to be public for this peer review?** For information about this choice, including consent withdrawal, please see our [Privacy Policy](https://www.plos.org/privacy-policy).

Reviewer #1: No

Reviewer #2: **Yes:** Rukhsana Ahmed

[NOTE: If reviewer comments were submitted as an attachment file, they will be attached to this email and accessible via the submission site. Please log into your account, locate the manuscript record, and check for the action link "View Attachments". If this link does not appear, there are no attachment files.]

**Reviewer #2:**

**Assessing malaria infection among pregnant women and children below five years of age attending rural health facilities of Kenya: A cross-sectional survey in two counties of Kenya**

Abstract

Background

In the main article (lines 121-124) you have stated the aim was “to determine the positivity rate of *Plasmodium falciparum* parasites in pregnant women and children below five years of age seeking healthcare services at the rural health facilities of Kwale and Siaya counties as well as their access and uptake of malaria control integrated services offered in those facilities”.

Suggest that you mention the same objectives in the abstract as well.

>> I have put these objectives in the abstract section as well, see lines 41-45.

Methods

The authors state in line 42 : “*cluster random sampling was used to select pregnant women and children…*.”. Suggest that the authors provide more information on how cluster allocation was done and how many clusters were there and number of women and children in each cluster. It would also help the reader if information is provided how clustering was considered for statistical analysis.

>> Thank you for this observation, we have now clarified this in the study design and sampling subsection, see lines 143-169. Additionally, we have stated that our analyses considered the study clusters, see lines 254-263.

>> However, due to limited word counts in the abstract section we cannot provide all these details in the abstract, but rather we have now made it clear in the pointed areas within the article.

Results

Lines 49-50, might be good to show within brackets the malaria positivity rate in Siaya Country. In showing the rates, the reader could get the sense for the high rate of malaria positivity.

>> Thanks for this comment, we have now put these rates in bracket, see line 56.

Also it is worthwhile to show the positivity rates detected by microscopy.

>> Thanks for this comment, we have now added this information, see lines 53-55.

Conclusion

You could strengthen your conclusion stating the importance of ANC attendance based on the fact of the higher risk of malaria infection in pregnant women who had not started ANC visits.

>> We have added this now, see lines 64-66.

Major Comments

Introduction

In the paragraph Lines 85-89, suggest to describe the malaria prevalence in pregnant women and in young children in Kenya showing prevalence figures.

>> Thanks for this suggestion, we have now added the description, please see lines 98-100.

Methods

In the paragraph under the sub-heading Study design and sampling (lines 128-137) suggest to include more details with attention to the following points:

Line 128-129: Expand the description of malaria status with reference to prevalence figures in the counties in Kwale and Siaya counties where you state it is moderate and high malaria endemic areas

>> We have now included these details, please see lines 158-160.

- Inclusion and exclusion criteria of pregnant women and children, i.e gestational age of pregnant women, trimester, parity that were considered for inclusion. For children, whether they were paired children of the pregnant women, or a separate sample, any age limit, conditions excluded.

>> We have now added a description of the inclusion criteria for both pregnant women and children below five years. Please note that we included pregnant women regardless of their gestational age (trimester) or parity.

- How cluster allocation was performed and the number of clusters and number of women and children in each cluster.

>> We have now explained what we meant by clusters and stated that equal number of participants (both children and pregnant women) were taken in each cluster.

- To lead the reader into the study please give more details of sample size calculation, instead of how it is stated currently in the sentence in line 136 “*The sample size calculation was based on previously documented specific malaria prevalence, for each specific group, in the said regions”*.

>> We have now provided more details on sample size calculation.

Sample processing and examination using microscopy ( Lines 153-157):

Since both thick and thin films were made, state what was the thick film used for and why thin film was preferred for determining parasitaemia and parasite density calculations?

>> Thick blood smear was used to detect infection and estimate low parasitaemia because the blood was concentrated hence allowed a greater volume of blood to be examined and malaria parasites were more concentrated as the RBCs are lysed. On the other hand, thin blood smear was examined to identify malaria species, quantify parasitaemia, and recognize parasites’ asexual forms because the smear is thinly spread, fixed using absolute methanol that allowed better visualization of the parasite. Please see lines 193-204 where we have stated the use of both thick and thin smears.

Please state how parasite negativity was determined and how was quality assurance of positive and negative reading managed.

>> All samples examined and observed to have no malaria parasite were regarded as negative result, please see lines 207-208.

>> We have added a statement on how quality was assured, see lines 208-212.

PCR analysis (Lines 166-183): Were the laboratory technicians and staff blind to microscopy results? If so, please state how blinding was done for laboratory analysis.

>> Yes, laboratory staff performing PCR analysis were blinded to the microscopy results since they received samples labelled with IDs only without any microscopy results. We now stated this in the manuscript, see lines 238-240.

Results

Suggest providing a baseline characteristic table of the study participants and reference it to what is described in the introductory paragraph to the results ( lines 201-209).

>> Thanks for the suggestion, however, our current table 1 contains all these information in the paragraph. We have referenced it appropriately.

Lines 210-211 a change of header to “ Malaria positivity rates by microscopy and PCR in the two study counties” might be more appropriate since what you describe is about the rates in the counties and later you are showing separately the malaria positivity rates in pregnant women and children.

>> Thanks for this suggestion, we have changed the heading appropriately.

Lines 215-218, Although you give reference to Table 1, it will help the reader if you mention whether it is PCR or microscopy results that are shown.

>> Thanks for this observation, we have now stated that these results refer to PCR results, see lines 285-291.

Line 281: Children below five years: did you consider to categorise the 0-5 years into age group and check for the risk of malaria in the age sub-groups. If you haven’t done so might be helpful to look into it see whether the odds of malaria changes in younger children below 2 yrs.

>> Thanks for this suggestion. We have now categorize the age of the children into sub-groups as indicated in tables 1 and 3. Further, we have explained the malaria risk by children age groups in the text, see lines 360-363.

Lines 287: Pregnant women and Table 3: suggest to include gravidity and show the results of the odds of malaria in different gravidae. It would help to see if there is any difference in the risk of malaria in different gravidae in the sub-counties with low malaria positivity rate.

>> Thanks for this additional suggestion, however we did not capture the number of past pregnancies a woman has had in her lifetime.

Discussion

Line 333: “malaria positivity rate was high among women aged, either 38 to 43 years or 14 to 19 years” the authors describe well on the point about higher malaria positivity rate in adolescents. Might be useful to explain the high positivity rate in this setting in the age group of pregnant women 38-43 years in light of gravidity and parity in addition to immunity.

>> Thanks for this comment, we have updated the explanation here, please see lines 417-423.

Line 352: “showed that age of the pregnant women was not significantly associated with malaria infection…”, this point is confusing and unclear, since earlier you have shown and described that adolescents and older women had higher malaria positivity. Try and explain this point and if in multivariate analysis age factor was not significant, why it might be not associated with malaria when adjusted for other factors.

>> Our analysis indicated that even though there was slightly higher malaria positivity rate among young pregnant women, a pregnant woman’s age group was not a significant risk factor in both univariable and multivariable models. So our points as explained in those paragraphs you have selected are not confusing.

Study Limitation

Line 358: “our results may then be biased by the exclusion of pregnant women who were not enrolled for antenatal care or deliver at home”. In your result section you have shown women who have not started ANC care and therefor this statement is contradictory. Please clarify these points better. Also through out the study there has been no mention about women and delivery or place of delivery and if it was an exclusion criteria please specify it when you show the eligibility criteria.

>> Thanks for this point. However, we don’t find any contradiction in this statement. We did not ask the women their place of delivery or a place they intend to deliver. In the eligibility criteria, we only enrolled women who came to ANC facility on the day of the survey and the visit for that day was not counted.

Table 5: It might be helpful if in the footnote the factors for which the results shown were adjusted is stated.

>> Sorry we don’t have table 5, maybe you are referring to table 4. If that is the case then all factors adjusted for this analysis are the ones within that table. Additionally, we have now improved on our explanation on how multivariable analysis was conducted, see lines 259-267.

Minor Comments

Line 246: “ A paltry 27 (8.2%) of the ANC attendees…..”. think you could rephrase this sentence as “Only 8.2% (27 women) of the ANC attendees had completed four ANC visits…..”

>> Thanks for this suggestion, we have rephrased appropriately.

Line 318: “similar results were observed by van Eijk and his colleagues [3]…” can Eijk is a female, and so this sentence should be rephrased “….van Eijk and her colleagues: if you not sure of the gender of the author you could write such sentence as “ van Eijk and colleagues…”.

>> Thank you very much for pointing out this oversight, we have corrected appropriately.
