# Supplementary material for: Assessment of malaria infection among pregnant women and children below five years of age attending rural health facilities of Kenya: A cross-sectional survey in two counties of Kenya
Source: PLoS One. 2021 Sep 16;16(9):e0257276. doi: 10.1371/journal.pone.0257276 (PMC8445417; doi:10.1371/journal.pone.0257276)
Supplement: Attachment — Submitted filename: Response to reviewers.docx [file pone.0257276.s005.docx]

PONE-D-20-23758R1
Assessing malaria infection among pregnant women and children below five years of age attending rural health facilities of Kenya: A cross-sectional survey in two counties of Kenya

**Comments to the Author**

1. If the authors have adequately addressed your comments raised in a previous round of review and you feel that this manuscript is now acceptable for publication, you may indicate that here to bypass the “Comments to the Author” section, enter your conflict of interest statement in the “Confidential to Editor” section, and submit your "Accept" recommendation.

Reviewer #1: (No Response)

Reviewer #2: All comments have been addressed

2. Is the manuscript technically sound, and do the data support the conclusions?

The manuscript must describe a technically sound piece of scientific research with data that supports the conclusions. Experiments must have been conducted rigorously, with appropriate controls, replication, and sample sizes. The conclusions must be drawn appropriately based on the data presented.

Reviewer #1: Partly

Reviewer #2: Yes

3. Has the statistical analysis been performed appropriately and rigorously?

Reviewer #1: No

Reviewer #2: Yes

4. Have the authors made all data underlying the findings in their manuscript fully available?

The [PLOS Data policy](http://www.plosone.org/static/policies.action#sharing) requires authors to make all data underlying the findings described in their manuscript fully available without restriction, with rare exception (please refer to the Data Availability Statement in the manuscript PDF file). The data should be provided as part of the manuscript or its supporting information, or deposited to a public repository. For example, in addition to summary statistics, the data points behind means, medians and variance measures should be available. If there are restrictions on publicly sharing data—e.g. participant privacy or use of data from a third party—those must be specified.

Reviewer #1: Yes

Reviewer #2: Yes

5. Is the manuscript presented in an intelligible fashion and written in standard English?

PLOS ONE does not copyedit accepted manuscripts, so the language in submitted articles must be clear, correct, and unambiguous. Any typographical or grammatical errors should be corrected at revision, so please note any specific errors here.

Reviewer #1: Yes

Reviewer #2: Yes

6. Review Comments to the Author

Please use the space provided to explain your answers to the questions above. You may also include additional comments for the author, including concerns about dual publication, research ethics, or publication ethics. (Please upload your review as an attachment if it exceeds 20,000 characters)

Reviewer #1: The authors have not fully addressed several key components of my prior comments. Below in the reiteration of some key points that make the manuscript, in my opinion, unacceptable for publication as it is currently written.

>> We have further responded satisfactorily to each of your concerns as below.

Below are: my prior comments, >>Authors: denotes the authors reply, and **denotes my current responses and attempts to provide further clarification. Comments threads are separated by ____

_______

1. The analysis of ANC utilization and SP-IPTp uptake should consider the trimester of the
participants. For example in Line 246: Was the 8.2% of ANC attendees who completed ANC
visits counted only from women late in the third trimester?

>> Authors: This was counted among all study-enrolled women, but women who were found to have completed their ANC visits were mostly in their third trimester.

**Women cannot have completed their ANC visits prior to the end of their third trimester. One cannot determine the completeness of their ANC care (number of ANC visits or number of IPTp doses) until delivery. This study design cannot answer this question unless you limit the analysis to women who are at full term. Women must either be followed throughout their pregnancies or must have retrospective data collection at the time of delivery.

>> Thanks for this follow-up question. We agree that with a cross-sectional study design, we cannot conclusively answer this question. However, please note that the study recruited pregnant women who were at various stages of their pregnancies with others who were about to deliver. So it is possible that we met participants who were at their last ANC visits (or possibly had completed the recommended four visits). We have therefore, amended this particular sentence to read as follows “…At the time of the study, 27 women (8.2%) of the ANC attendees reported to have completed four ANC attendance, despite the fact that they were yet to reach the full-term of the pregnancy….”, see lines 310-312. Additionally, we have amended related sections as can be seen in lines 321-323 and 406-409, and acknowledged this limitation in lines 452-457.

a. If women are in their first or second trimester they would not be expected to have 4 ANC visits completed. Nor should women in the first trimester have received SP-IPTp.

>> Authors: It is true that women in first or second trimester would not be expected to have complete
ANC visits, but the idea here was to find out at what stage of pregnancy do women start ANC
visits and also start receiving IPTp-SP doses and compare this with the national guidelines.

**I agree you may answer the trimester or gestational age of ANC enrollment with this study design, but as above the completeness of ANC care cannot be assessed in this study unless you only analyze full term women.

>> Thanks for this additional question on the completeness of the ANC attendance. We have elaborately responded to this issue in our above response (#1) and also acknowledged this limitation in lines 452-457.

b. It is also not clear if women were recruited from the ANC clinic how could any of the
participants could not be enrolled in antenatal care? This only seems possible if the study visit
took place prior to the clinic visit and the visit that day was not included. One way to clarify this
might be to stratify the current Table 2 by trimester of the woman enrolled. How to take this into
account in the univariate and multivariable analyses should also be considered (see #3).

>> Authors: Women were recruited immediately they arrived at the facility and prior to being attended to at the clinic, see lines 190-191. The ANC visit that day was not counted.

**Again, if women have come for antenatal care and you enroll them in your study you cannot count them as not having enrolled in antenatal care. If some of your participants did not follow through and complete the ANC visit they came to the clinic to receive, then that is a significant problem with the study. Based on the study design, you have systematically missed women who did not enroll in the ANC care.

>> Participants were coming to the health facility to receive a myriad of services, ANC being one of them. We recruited participants as they entered the facility provided they met the inclusion criteria, and as we had indicated, the visit that day was not counted. In our limitations, we had acknowledged that the study might have missed some women who were not enrolled for the ANC service.

**The results section on ANC attendance requires major revision and should be removed other than analyses of the timing of enrollment in antenatal care – with acknowledgement that this analysis is only among women who have presented for antenatal care and does not reflect the overall population.

>> We wish to disagree with your opinion that these results on ANC should be removed. In our opinion, these results are important to policy implementers since they indicate the variability of ANC attendance/IPTp uptake in the two rural communities, the timings of these visits/IPTp uptake and their relationship with malaria infection. Please note that we did a descriptive cross-sectional study with no follow-up surveys till the delivery period of the pregnancy (a limitation we have acknowledged and hence the readers will be aware of this limitation).

________

9. Methods: Pf infection is defined as requiring both positive microscopy and PCR. However,
much of the statistical analysis uses PCR positivity as the outcome. Consider clarifying the
definitions of infection in the methods. Based on the data in presented in Table 1, there were
many microscopy false positives or pcr false negatives (microscopy positivity > pcr positivity)
and as expected some submicroscopic infection (pcr positivity > being both pcr and microscopy
positive). Given that the results are primarily based on PCR, I assume the authors are more
confident in their PCR data. If so, they should be more clear about their interpretation of their
results and not leave the reader to make their own conclusions.

>> Authors: Thanks for this comment. We had stated that Pf infection was first defined by microscopy then confirmed by PCR (see lines 251-253). As much as many of the malaria results in the text were referring to PCR results we had also stated and compared it with microscopy both in the text and tables. We have now put more clarification here, please see lines 253-254. Additionally, we have now outlined the limitations of microscopy technique that made us compared its results with PCR and subsequently relied more on PCR results, see lines 469-478.

**The fact that microscopy prevalence is higher than PCR still raises concern. Your definition of infection requiring PCR confirmation of microscopy makes sense as you state the in the methods. However, then the analyses should be done using that as the outcome, e.g. Tables 3 and 4 should have the outcome as confirmed infection (microscopy and PCR positive) as compared to PCR only. Or you should decide that you trust your PCR and that PCR is truly detecting some submicroscopic infection. That can be justified and used. The key is to state your definition, justify it, and then use it.

>> Thanks for this additional comment. We have now provided justification for relying on the PCR results (see lines 244-245), and also stated in the methods that risk factors analysis was performed on PCR results, see line 248.

**The explanation in the Discussion of why routine health center microscopy may not be acurate is true. However, the methods section describes research grade microscopy – well trained technicians, double reading, support from a research facility. Thus, the description in the Discussion doesn’t fit the current microscopy results.

>> In the methodology section, we stated the exact survey procedures we followed in conducting this study, while in the discussion section (specifically the limitations part) we stated the known limitations of performing a microscopy test and at the end stated how we mitigated these known limitations (please see lines 464-469). We therefore don’t clearly understand what you mean by saying “…Thus, the description in the Discussion doesn’t fit the current microscopy results…” .

_______

4. Methods: How were the women and children chosen? Were they pairs, meaning the pregnant
women were there with an older child and so they were sampled together? If this were the case,
primigravidae would be underrepresented or absent. Also because of natural birth spacing,
younger children would be underrepresented.

>> Authors: The study did not pair pregnant women with children below five years. These were separate groups of participants who were coming to the health facility. But it was possible that some pregnant women were accompanied with their children who were below five years to the clinic. However, their data were not paired. We have now clarified this in the text, please see lines 188-
190.

**How the children were selected remains unclear. The methods state “children below five years of age attending ANC”. Were these children accompanying women to their ANC visit? Were they coming to immunization/other well care OR were they coming for sick visits? If they were coming for sick visits, then one might anticipate that the test positivity rate would be higher among a symptomatic population.

>> Thanks for this further comment. As we had stated, all participants, including children, were coming to a health facility (i.e. either a health centre, dispensary or mobile clinic) which was offering a myriad of services; ANC and child welfare services included. Among the participants we were interested in were children below 5 years seeking any of these services at the facility (including those who were merely accompanying their mothers/guardians to the facility), and these children were selected provided they met our stated inclusion criteria.

Reviewer #2: All comments from previous review has been adequately addressed. I have no further comments. The manuscript is acceptable for publication from my side.

>> Thanks for endorsing our earlier responses to your queries.
