# Supplementary material for: Assessment of malaria infection among pregnant women and children below five years of age attending rural health facilities of Kenya: A cross-sectional survey in two counties of Kenya
Source: PLoS One. 2021 Sep 16;16(9):e0257276. doi: 10.1371/journal.pone.0257276 (PMC8445417; doi:10.1371/journal.pone.0257276)
Supplement: Attachment — Submitted filename: Response to reviewers.docx [file pone.0257276.s006.docx]

PONE-D-20-23758R2
Assessment of malaria infection among pregnant women and children below five years of age attending rural health facilities of Kenya: A cross-sectional survey in two counties of Kenya
PLOS ONE

Dear Dr. Okoyo,

Thank you for submitting your manuscript to PLOS ONE. After careful consideration, we feel that only part of your manuscript merits further consideration.  The ANC completion and IPTp coverage information presented appears to be invalid for reasons of study design and queried by peer-review.  These cannot be overcome.  However, if you would like to have your manuscript further considered by PLOS ONE, kindly remove the ANC completion and IPTp coverage information entirely - text and table content - and submit a revised version.

>> Thanks for the opportunity to revise our manuscript further, we have now removed completely all the results related to ANC and IPTp completion. We believe now that all the comments that were raised are fully addressed.

Please submit your revised manuscript by Aug 08 2021 11:59PM. If you will need more time than this to complete your revisions, please reply to this message or contact the journal office at plosone@plos.org. When you're ready to submit your revision, log on to https://www.editorialmanager.com/pone/ and select the 'Submissions Needing Revision' folder to locate your manuscript file.

Please include the following items when submitting your revised manuscript:

- A rebuttal letter that responds to each point raised by the academic editor and reviewer(s). You should upload this letter as a separate file labeled 'Response to Reviewers'.
- A marked-up copy of your manuscript that highlights changes made to the original version. You should upload this as a separate file labeled 'Revised Manuscript with Track Changes'.
- An unmarked version of your revised paper without tracked changes. You should upload this as a separate file labeled 'Manuscript'.

If you would like to make changes to your financial disclosure, please include your updated statement in your cover letter. Guidelines for resubmitting your figure files are available below the reviewer comments at the end of this letter.

If applicable, we recommend that you deposit your laboratory protocols in protocols.io to enhance the reproducibility of your results. Protocols.io assigns your protocol its own identifier (DOI) so that it can be cited independently in the future. For instructions see: <http://journals.plos.org/plosone/s/submission-guidelines#loc-laboratory-protocols>. Additionally, PLOS ONE offers an option for publishing peer-reviewed Lab Protocol articles, which describe protocols hosted on protocols.io. Read more information on sharing protocols at <https://plos.org/protocols?utm_medium=editorial-email&utm_source=authorletters&utm_campaign=protocols>.

We look forward to receiving your revised manuscript.

Kind regards,

R Matthew Chico, MPH, PhD
Academic Editor
PLOS ONE

**Comments to the Author**

Reviewer #1: The authors have not addressed key components of my prior comments. Again these issues make the manuscript, in my opinion, unacceptable for publication as it is currently written. In this revision the authors have acknowledged the limitations of some of their analyses, but the misleading analyses remain in the abstract and the results sections.

My prior reviews have described the rationale for why some of the presented analyses are misleading. My recommendation is that these sections of the manuscript should be removed rather than discounted in the discussion. Specifically at least the following statements should be removed.

Abstract:
“Of all the pregnant women, only 27 (8.2%) had attained the recommended number of ANC attendance of at least four visits and only 55 (13.2%) had received the recommended number of intermittent preventive treatment with sulfadoxine-pyrimethamine (IPTp-SP) of at least three doses.”

>> We have now removed this statement entirely in the abstract section.

Results:
“At the time of the study, only 27 women (8.2%) of the ANC attendees reported to have completed four ANC attendance, despite the fact that they were yet to reach the full-term of the pregnancy.”
>> We have now removed this statement entirely in the results section.

“However, at the time of the survey, in overall, only a total of 55 (11.6%) of the pregnant women had received the recommended dosage of three or more doses of IPTp-SP (again noting that all these women were yet to deliver).”

>> We have now removed this statement entirely in the results section.


Many of the same conclusions can be reached by focusing on results showing that many women enroll in ANC late and thus do not benefit from clearing infections early in pregnancy: “Additionally, many of the pregnant women surveyed made their first ANC visit in their second trimester, 119 (37.0%), and third trimester, 200 (62.1%).” And “majority of the women got their first dose (of SP) between the sixth (18.7%) and eighth (25.3%) months.”

My prior comments on other aspects of the manuscript that remain unaddressed are below, though the most critical component is above.
>> We believe that we have now fully addressed these critical comments.


--------------

1. It is also not clear if women were recruited from the ANC clinic how could any of the
participants could not be enrolled in antenatal care? This only seems possible if the study visit
took place prior to the clinic visit and the visit that day was not included. One way to clarify this
might be to stratify the current Table 2 by trimester of the woman enrolled. How to take this into
account in the univariate and multivariable analyses should also be considered (see #3).

>> Authors: Women were recruited immediately they arrived at the facility and prior to being attended to at the clinic, see lines 190-191. The ANC visit that day was not counted.

**Revision 1: Again, if women have come for antenatal care and you enroll them in your study you cannot count them as not having enrolled in antenatal care. If some of your participants did not follow through and complete the ANC visit they came to the clinic to receive, then that is a significant problem with the study. Based on the study design, you have systematically missed women who did not enroll in the ANC care.

**Revision 1:The results section on ANC attendance requires major revision and should be removed other than analyses of the timing of enrollment in antenatal care – with acknowledgement that this analysis is only among women who have presented for antenatal care and does not reflect the overall population.

**Revision 2: “Pregnant women who had not started ANC visits were up to five times at risk of malaria infection, (adjusted odds ratio = 5.40, 95%CI: 0.96-30.50, p=0.046).” In fact these are women coming for their first ANC visit. These data do not include women who were in the community and not sought ANC care. Phrasing this as “Pregnant women presenting for their first ANC visit had up to five times higher odds of infection (aOR…) compared to women who had completed >= 4 ANC visits” is more accurate.
>> We have rephrased this statement and all the other related statements in the text.

2. Methods: Pf infection is defined as requiring both positive microscopy and PCR. However,
much of the statistical analysis uses PCR positivity as the outcome. Consider clarifying the
definitions of infection in the methods. Based on the data in presented in Table 1, there were
many microscopy false positives or pcr false negatives (microscopy positivity > pcr positivity)
and as expected some submicroscopic infection (pcr positivity > being both pcr and microscopy
positive). Given that the results are primarily based on PCR, I assume the authors are more
confident in their PCR data. If so, they should be more clear about their interpretation of their
results and not leave the reader to make their own conclusions.

>> Authors: Thanks for this comment. We had stated that Pf infection was first defined by microscopy then confirmed by PCR (see lines 251-253). As much as many of the malaria results in the text were referring to PCR results we had also stated and compared it with microscopy both in the text and tables. We have now put more clarification here, please see lines 253-254. Additionally, we have now outlined the limitations of microscopy technique that made us compared its results with PCR and subsequently relied more on PCR results, see lines 469-478.

**Revision 1: The fact that microscopy prevalence is higher than PCR still raises concern. Your definition of infection requiring PCR confirmation of microscopy makes sense as you state the in the methods. However, then the analyses should be done using that as the outcome, e.g. Tables 3 and 4 should have the outcome as confirmed infection (microscopy and PCR positive) as compared to PCR only. Or you should decide that you trust your PCR and that PCR is truly detecting some submicroscopic infection. That can be justified and used. The key is to state your definition, justify it, and then use it.

**Revision 1: The explanation in the Discussion of why routine health center microscopy may not be acurate is true. However, the methods section describes research grade microscopy – well trained technicians, double reading, support from a research facility. Thus, the description in the Discussion doesn’t fit the current microscopy results.
>> Please note that what we have put in the discussion about microscopy are the generally-known limitations of performing microscopy procedure that makes this technique less reliable. However, what we have put on the methods are the actual steps we followed in performing our microscopy procedure for this study. Thus, the results described here are as per the procedure described in the methods.

>> We would appreciate a specific suggestion on what you would like to see regarding our outline of the microscopy procedure that we performed in the field.

3. Methods: How were the women and children chosen? Were they pairs, meaning the pregnant
women were there with an older child and so they were sampled together? If this were the case,
primigravidae would be underrepresented or absent. Also because of natural birth spacing,
younger children would be underrepresented.

>> Authors: The study did not pair pregnant women with children below five years. These were separate groups of participants who were coming to the health facility. But it was possible that some pregnant women were accompanied with their children who were below five years to the clinic. However, their data were not paired. We have now clarified this in the text, please see lines 188-
190.

**Revision 1:How the children were selected remains unclear. The methods state “children below five years of age attending ANC”. Were these children accompanying women to their ANC visit? Were they coming to immunization/other well care OR were they coming for sick visits? If they were coming for sick visits, then one might anticipate that the test positivity rate would be higher among a symptomatic population.

>> In line 185-186, we had indicated that each group of participants “…separately came to the facility to seek services.” Some of these services the children came to seek could be immunization, post-natal check-ups, other forms of treatments etc, as well as simply accompanying their pregnant mothers/guardians.

>> Additionally, we have now explained some of the possible reasons why children were coming to the facility, please see lines 162-165.
