# Supplementary material for: Assessment of malaria infection among pregnant women and children below five years of age attending rural health facilities of Kenya: A cross-sectional survey in two counties of Kenya
Source: PLoS One. 2021 Sep 16;16(9):e0257276. doi: 10.1371/journal.pone.0257276 (PMC8445417; doi:10.1371/journal.pone.0257276)
Supplement: Attachment — Submitted filename: R.Ahmed_Reviewer comments.docx [file pone.0257276.s007.docx]

**Assessing malaria infection among pregnant women and children below five years of age attending rural health facilities of Kenya: A cross-sectional survey in two counties of Kenya**

Author: We note that these review comments seemed to have been raised earlier and were adequately addressed in previous review responses.

Abstract

Background

In the main article (lines 121-124) you have stated the aim was “to determine the positivity rate of *Plasmodium falciparum* parasites in pregnant women and children below five years of age seeking healthcare services at the rural health facilities of Kwale and Siaya counties as well as their access and uptake of malaria control integrated services offered in those facilities”.

Suggest that you mention the same objectives in the abstract as well.

>> This was already stated in the abstract as well, please see lines 40-43.

Methods

The authors state in line 42 : “*cluster random sampling was used to select pregnant women and children…*.”. Suggest that the authors provide more information on how cluster allocation was done and how many clusters were there and number of women and children in each cluster. It would also help the reader if information is provided how clustering was considered for statistical analysis.

>> Details about cluster random sampling are provided in the methods section of the manuscript. Abstract section is limited by the required word counts.

>> We specifically answer your question by breaking it down as follows:

How cluster allocation was done: Cluster allocation was done randomly, see lines 143-145.

How many clusters: Clusters were defined as health facilities and therefore we included 5 clusters in Kwale County and 6 clusters in Siaya County, see lines 143-145.

Number of women and children per cluster: Sample size was specifically calculated for each county (i.e. according to the region’s malaria endemicity). Then the resulting sample size was equally allocated to each cluster, see lines 145-146.

How was clustering considered in the statistical analysis: All our statistical analyses were adjusted for clustering by the health facilities (clusters) as indicated in the analysis section, see lines 248-257.

Results

Lines 49-50, might be good to show within brackets the malaria positivity rate in Siaya Country. In showing the rates, the reader could get the sense for the high rate of malaria positivity.

>> We have compared the rates for both Siaya and Kwale counties for both groups of participants and different test techniques, please see lines 53-57.

Also it is worthwhile to show the positivity rates detected by microscopy.

>> We indeed mentioned these microscopy rates, please see lines 49-57.

Conclusion

You could strengthen your conclusion stating the importance of ANC attendance based on the fact of the higher risk of malaria infection in pregnant women who had not started ANC visits.

This had been addressed, see lines 467-476

Major Comments

Introduction

In the paragraph Lines 85-89, suggest to describe the malaria prevalence in pregnant women and in young children in Kenya showing prevalence figures.

>> These prevalence figures had been given in lines 92-94.

Methods

In the paragraph under the sub-heading Study design and sampling (lines 128-137) suggest to include more details with attention to the following points:

Line 128-129: Expand the description of malaria status with reference to prevalence figures in the counties in Kwale and Siaya counties where you state it is moderate and high malaria endemic areas

- Inclusion and exclusion criteria of pregnant women and children, i.e gestational age of pregnant women, trimester, parity that were considered for inclusion. For children, whether they were paired children of the pregnant women, or a separate sample, any age limit, conditions excluded.

>> These had been addressed, please see lines 156-165.

- How cluster allocation was performed and the number of clusters and number of women and children in each cluster.

>> These had been addressed, please see lines 141-148.

- To lead the reader into the study please give more details of sample size calculation, instead of how it is stated currently in the sentence in line 136 “*The sample size calculation was based on previously documented specific malaria prevalence, for each specific group, in the said regions”*.

>> Details for the calculation of the sample size had been outlined in lines 148-156.

Sample processing and examination using microscopy ( Lines 153-157):

Since both thick and thin films were made, state what was the thick film used for and why thin film was preferred for determining parasitaemia and parasite density calculations?

>> This had been addressed in lines 189-194.

Please state how parasite negativity was determined and how was quality assurance of positive and negative reading managed.

>> This had been addressed in lines 203-208.

PCR analysis (Lines 166-183): Were the laboratory technicians and staff blind to microscopy results? If so, please state how blinding was done for laboratory analysis.

Results [All these comments had been addressed in the earlier revisions]

Suggest providing a baseline characteristic table of the study participants and reference it to what is described in the introductory paragraph to the results ( lines 201-209).

Lines 210-211 a change of header to “ Malaria positivity rates by microscopy and PCR in the two study counties” might be more appropriate since what you describe is about the rates in the counties and later you are showing separately the malaria positivity rates in pregnant women and children.

Lines 215-218, Although you give reference to Table 1, it will help the reader if you mention whether it is PCR or microscopy results that are shown.

Line 281: Children below five years: did you consider to categorise the 0-5 years into age group and check for the risk of malaria in the age sub-groups. If you haven’t done so might be helpful to look into it see whether the odds of malaria changes in younger children below 2 yrs.

Lines 287: Pregnant women and Table 3: suggest to include gravidity and show the results of the odds of malaria in different gravidae. It would help to see if there is any difference in the risk of malaria in different gravidae in the sub-counties with low malaria positivity rate.

Discussion

Line 333: “malaria positivity rate was high among women aged, either 38 to 43 years or 14 to 19 years” the authors describe well on the point about higher malaria positivity rate in adolescents. Might be useful to explain the high positivity rate in this setting in the age group of pregnant women 38-43 years in light of gravidity and parity in addition to immunity.

Line 352: “showed that age of the pregnant women was not significantly associated with malaria infection…”, this point is confusing and unclear, since earlier you have shown and described that adolescents and older women had higher malaria positivity. Try and explain this point and if in multivariate analysis age factor was not significant, why it might be not associated with malaria when adjusted for other factors.

Study Limitation

Line 358: “our results may then be biased by the exclusion of pregnant women who were not enrolled for antenatal care or deliver at home”. In your result section you have shown women who have not started ANC care and therefor this statement is contradictory. Please clarify these points better. Also through out the study there has been no mention about women and delivery or place of delivery and if it was an exclusion criteria please specify it when you show the eligibility criteria.

Table 5: It might be helpful if in the footnote the factors for which the results shown were adjusted is stated.

Minor Comments

Line 246: “ A paltry 27 (8.2%) of the ANC attendees…..”. think you could rephrase this sentence as “Only 8.2% (27 women) of the ANC attendees had completed four ANC visits…..”

>> This comment had been addressed in the previous reviews.

Line 318: “similar results were observed by van Eijk and his colleagues [3]…” can Eijk is a female, and so this sentence should be rephrased “….van Eijk and her colleagues: if you not sure of the gender of the author you could write such sentence as “ van Eijk and colleagues…”.

>> This had been addressed in line 381.
